# Effect of Atorvastatin combined with Irbesartan in the treatment of early diabetic nephropathy

**DOI:** 10.12669/pjms.40.7.9214

**Published:** 2024-08

**Authors:** Xiaojing Zhong, Jiajia Fan

**Affiliations:** 1 Xiaojing Zhong, Department of Endocrinology, Huzhou Central Hospital, 1558 Sanhuan North Road, Huzhou, Zhejiang Province 313003, P.R. China; 2 Jiajia Fan, Department of General Medicine, Huzhou Central Hospital, 1558 Sanhuan North Road, Huzhou, Zhejiang Province 313003, P.R. China

**Keywords:** Atorvastatin, Irbesartan, Early diabetic nephropathy

## Abstract

**Objective:**

To explore the effect of Atorvastatin combined with Irbesartan in the treatment of early diabetic nephropathy (DN).

**Methods:**

Clinical data from 153 patients with early DN, admitted to Huzhou Central Hospital from January 2020 to December 2022, was retrospectively selected. Patients were divided into two groups based on the treatment they received: patients received Irbesartan treatment alone were assigned to Irbesartan group (n=74); patients received Irbesartan combined with Atorvastatin were assigned to combined group (n=79). Levels of renal function indicators, renal fibrosis indicators, micro inflammatory status indicators, and incidence of adverse reactions were compared between the two groups before and after the treatment.

**Results:**

After the treatment, indicators of renal function, renal fibrosis and micro-inflammation in both groups significantly decreased compared to pretreatment levels (*P*<0.05), and were significantly lower in the combined group compared to Irbesartan group (*P*<0.05). There was no significant difference in the incidence of adverse reactions between the groups (*P*>0.05).

**Conclusions:**

Compared with Irbesartan alone, Atorvastatin combined with Irbesartan is more effective in the treatment of early DN. Combined treatment regimen is able to effectively reduces the micro-inflammatory state, improve renal function and fibrosis, and is not associated with the increased risk of adverse reactions.

## INTRODUCTION

Diabetic nephropathy (DN) is a common complication in patients with diabetes, and about 20% of people with type 2 diabetes develop symptoms of DN within 20 years of onset of diabetes.^1^ Its pathogenesis is closely related to abnormalities in glomerular dynamics, disorders of the coagulation-fibrinolysis system, oxidative stress, and changes in glucose and lipid metabolism.^1^,^2^ Patients with DN that do not receive timely and effective intervention, as the condition progresses, it will eventually lead to end-stage kidney disease.^3^,^4^

Irbesartan an angiotensin inhibitor that can regulate proteinuria and glomerular internal pressure, protect the kidney, and inhibit disease progression, is commonly used for treating kidney diseases.^4^,^5^ Li *et al*.^6^ showed that Irbesartan has high fat solubility and good tissue penetration, and can selectively block the binding between angiotensin II and receptors, inhibit the secretion of aldosterone, selectively expand the efferent arteriole, correct the hemodynamic changes in patients with DN, reduce UAER, and alleviate kidney damage.

Recent studies show that hyperlipidemia is a major determinant of progression of renal disease in patients with diabetes,^7^ and, atorvastatin, a frequently used lipid-lowering drug in clinic, can also effectively reduce proteinuria, protect the kidney, and improve renal function.^8^,^9^ Fotso *et al*.^10^ pointed out that atorvastatin has a lipid-independent renal protection effect, can restore the function of vascular endothelial cells, and inhibit the proliferation of mesangial cells by downregulating the expression of cytokines, thus inhibiting the progression of DN.

However, there is still not enough studies of the safety and efficiency of atorvastatin combined with Irbesartan for the treatment of patients with early DN. We performed a retrospective analysis of patients with early DN who received atorvastatin combined with Irbesartan in our hospital to assess the efficiency of the combined regimen in the treatment of early DN. Our results may provide practical reference for relevant clinical treatment.

## METHODS

Medical records of 153 patients with early DN (79 males and 74 females) who received treatment in Huzhou Central Hospital from January 2020 to December 2022 were collected. Age of the patients ranged from 45 to 83 years, with an average age of 64.15 ± 8.82 years. The course of diabetes ranged from 3 to 20 years, with an average of 10.42 ± 4.01 years. Of 153 patients, 74 were treated with Irbesartan alone were set as Irbesartan group, and 79 received Atorvastatin combined with Irbesartan were set as the combined group.

### Ethical Approval:

The Ethics Committee of our hospital approved this study with the Ref. No. 2023041201 on April 12^th^ 2023.

### Inclusion criteria:

- Patients diagnosed as DN.^11^
- The clinical data is complete.

### Exclusion criteria:

- Patients with acute or chronic inflammation.
- Patients who have used angiotensin receptor blocker (ARB), angiotensin-converting enzyme inhibitors (ACEI), and lipid-lowering drugs.
- Presence of fever and infection.
- Narrow renal arteries, acute and chronic nephritis and other causes of proteinuria.
- Patients with benign and malignant tumors.

### Treatment:

Both groups of patients with early DN were treated with symptomatic basic interventions such as reducing blood pressure, lowering blood sugar, enhancing exercise and adjusting diet. Patients in the Irbesartan group received Irbesartan (Hangzhou Sanofi Pharmaceutical Co., Ltd., Approval No.: H20040494; 0.15g/tablet) alone. It was administered orally once a day, one tablet/time. Patients in the combined group received Atorvastatin (Pfizer Pharmaceutical Co., Ltd.; Approval No.: H20051407; specification: 10 mg × 7 tablets) on the basis of Irbesartan. Atorvastatin was taken orally once a day, two tablets per time. Both groups were treated continuously for three months.

### Outcome measures:

- Baseline data including sex, age, course of diabetes, blood pressure, fasting blood glucose (FBG) and urinary microalbumin (mALB) levels. Blood pressure was measured using a fully automatic electronic blood pressure meter (Model: ZS-15-A; Dongbei Medical; Shandong, China). FBG was detected by hexokinase method. Briefly, patients were fasting for at least eight hours before testing, and the blood glucose levels in the venous blood serum was measured using the Hitachi 7600 automatic biochemical analyzer (Hitachi, Japan). mALB was detected using the Beckman Array360 fully automatic specific protein analyzer (Beckman Coulter Inc., Brea, CA, USA) and matching reagent kit.
- Renal function indicators, such as UAER, blood urea nitrogen (BUN) and serum creatinine (Scr) levels. UAER was detected by radioimmunoassay kit (Chinese Nuclear Institute, Beijing, China), Scr and BUN were measured by an automatic biochemical analyzer (Olympus AU 2700, USA).
- Renal fibrosis indicators including the levels of connective tissue growth factor (CTGF), tissue inhibitor of matrix metalloproteinase-1 (TIMP-1), and transforming growth factor-β1 (TGF-β1) were detected by enzyme-linked immunosorbent assay; The reagent kit was purchased from Beijing Furui Biotechnology Co., Ltd.
- Microinflammatory status indicators such as tumor necrosis factor-α (TNF-α), interleukin-1 (IL-1), interleukin 6 (IL-6) were measured by enzyme-linked immunosorbent assay using the reagent kit from Beijing Furui Biotechnology Co., Ltd.
- The incidence of adverse reactions was also recorded.

### Statistical Analysis:

All data analysis was conducted using SPSS26.0 software (IBM Corp, Armonk, NY, USA). The normality of the data was evaluated using the Shapiro Wilk test. The data of normal distribution were expressed as mean ± standard deviation. Independent sample t-test was used for inter-group comparison. Paired t-tests were used for intra-group comparison before and after the treatment. The counting data were represented by the number of cases, and Chi-squared test was used for comparison between groups. P<0.05 was considered statistically significant.

## RESULTS

A total of 153 patients were included in this retrospective study. There were 74 patients in the Irbesartan group and 79 patients in the combined group, with no significant difference in the baseline data between the groups (P>0.05). Table-I.

**Table-I:** Comparison of baseline data between two groups.

Basic information	Combined group (n=79)	Irbesartan group (n=74)	χ^2^/t	p-Value
Gender (male/female)	38/41	41/33	0.816	0.366
Age (years)	64.61±8.39	63.66±9.28	0.662	0.509
Course of diabetes (years)	10.03±3.53	10.84±4.45	-1.245	0.215
Systolic blood pressure (mmHg)	126.73±11.59	129.70±11.36	-1.599	0.112
Diastolic blood pressure (mmHg)	68.66±8.65	67.80±7.10	0.670	0.504
FBG (mmol/L)	5.81±1.03	5.52±0.89	1.864	0.064
mALB (ng/g·Cr)	72.15±10.24	70.64±9.90	0.930	0.354

Before the treatment, there was no significant difference in the levels of renal function indicators between the two groups (P>0.05). After the treatment, the levels of UAER, BUN, and Scr in both groups significantly decreased compared to pretreatment levels, and were significantly lower in the combination group compared to the Irbesartan group (P<0.05). Table-II

**Table-II:** Comparison of renal function indicators between two groups.

Time	Group	n	UAER (ug/min)	BUN (mmol/L)	Scr (umol/L)
Before treatment	Combined group	79	123.67±17.04	7.16±0.95	155.48±25.40
	Irbesartan group	74	120.08±19.94	7.31±1.09	151.28±23.24
	*t*		1.199	-0.892	1.064
	*P*		0.232	0.374	0.289
After treatment	Combined group	79	60.80±13.14^a^	5.98±0.90^a^	112.54±22.41^a^
	Irbesartan group	74	73.28±11.70^a^	6.60±1.11^a^	126.61±20.17^a^
	*t*		-6.193	-3.781	-4.071
	*P*		<0.001	<0.001	<0.001

***Note:*** Compared with the same group before treatment,

a P<0.05.

Before the treatment, there levels of renal fibrosis indicators were comparable in both groups (P>0.05). After three months of treatment, the levels of CTGF, TIMP-1, and TGF-β1 in both groups significantly decreased overall, and were significantly lower in the combination group compared to the Irbesartan group (P<0.05). Table-III

**Table-III:** Comparison of renal fibrosis indicators between two groups.

Time	Group	n	CTGF (ug/L)	TIMP-1 (ng/ml)	TGF-β1 (ng/ml)
Before treatment	Combined group	79	41.10±6.53	1333.04±237.13	168.70±20.95
	Irbesartan group	74	43.04±7.13	1375.51±254.35	173.39±17.68
	*t*		-1.756	-1.069	-1.493
	*P*		0.081	0.287	0.137
After treatment	Combined group	79	23.78±5.00^a^	784.78±122.37^a^	110.18±19.16^a^
	Irbesartan group	74	27.23±5.47^a^	987.59±184.31^a^	130.30±17.39^a^
	*t*		-4.070	-7.963	-6.785
	*P*		<0.001	<0.001	<0.001

***Note:*** Compared with the same group before treatment,

a P<0.05.

Before the treatment, there was no significant difference in the levels of micro-inflammatory indicators between the two groups (P>0.05). After three months of treatment, the levels of TNF-α, IL-1, and IL-6 in both groups significantly decreased compared to pretreatment levels, and were significantly lower in the combination group than in the Irbesartan group (P<0.05). Table-IV. There was no significant difference in the incidence of adverse reactions between the combination group (5.06%) and the Irbesartan group (2.70%) (P>0.05). Table-V

**Table-IV:** Comparison of microinflammatory index levels between two groups.

Time	Group	n	TNF-α (ng/ml)	IL-1 (pg/ml)	IL-6 (ng/L)
Before treatment	Combined group	79	38.33±5.18	19.75±4.49	22.16±3.28
	Irbesartan group	74	37.74±5.03	18.93±4.59	21.28±3.89
	t		0.709	1.110	1.517
	*P*		0.479	0.269	0.131
After treatment	Combined group	79	20.29±4.97^a^	11.44±4.39^a^	11.76±3.07^a^
	Irbesartan group	74	25.69±4.02^a^	14.43±4.62^a^	14.45±3.13^a^
	*t*		-7.355	-4.104	-5.366
	*P*		<0.001	<0.001	<0.001

***Note:*** Compared with the same group before treatment,

a P<0.05.

**Table-V:** Comparison of adverse reactions between two groups.

Group	n	Dizzy	Gastrointestinal discomfort	Vomit	Total occurrence rate
Combined group	79	1 (1.27)	2 (2.53)	1 (1.27)	4 (5.06)
Irbesartan group	74	0 (0.00)	1 (1.35)	1 (1.35)	2 (2.70)
*χ^2^*					0.565
*P*					0.452

## DISCUSSION

The results of this study show that compared to Irbesartan monotherapy, a combined regimen of atorvastatin and Irbesartan is safe and can more effectively improve renal function and renal fibrosis indicators and lower inflammatory reaction in patients with early DN.

Zhao *et al*.^12^ showed that irbesartan monotherapy of DN can alleviate the clinical symptoms of patients to a certain extent, but the overall effect is still below the expected clinical level. Zuo *et al*.^13^ confirmed that Irbesartan combined with atorvastatin was more effective in improving renal function and blood lipid indicators of patients with DN that irbesartan alone. Consistently with these reports, our results further confirm that the combined medication regimen has higher feasibility and efficiency in improving patient’s renal function. However, Zuo *et al*.^13^ showed a significant increase in the incidence of nausea and vomiting in patients who received a combined treatment. The discrepancy between this observation and our results may be related to factors such as patient tolerance. Therefore, further clinical follow-up studies are needed to assess the safety of a combined regimen.

Liu *et al*.^14^ treated patients with DN with Irbesartan alone or in combination with a dipeptidyl peptidase-4 (DPP-4) inhibitor, linagliptin. The results showed that the combined treatment led to significantly improved levels of FBG, 2hPBG, HbA1C, Cys-C, SCr, BUN and UACR compared to patients in the single drug group, with no increase in the incidence of serious adverse reactions. This is consistent with the results of this study. Li *et al*.^15^ confirmed that the addition of atorvastatin to the conventional treatment can effectively regulate the hemorheology state and reduce the degree of renal function damage in patients with DN. Studies have showed that inflammation plays an important role in the pathogenesis and progression of DN. TNF-α, a polypeptide cytokine secreted by mesangium cells,^16^ can increase the levels of inflammatory factors, eventually leading to kidney damage.^16^ IL-1α and IL-1α, that are mainly generated by activated macrophages, are important regulatory factors in inflammatory response.^16^ IL-6 can play a role in vascular endothelial cells, promoting thrombosis, enhancing vascular permeability, and exacerbating the progression of DN.^16^,^17^

Moreover, IL-6 can induce the expression and activity of proinflammatory hsCRP *in vivo*, which is closely related to the occurrence and progress of insulin resistance and various complications of diabetes. It may also cause abnormal oxidation and thickening of the mesangium, and cell proliferation, thus damaging the renal function of patients.^17^,^18^ Previous studies have demonstrated that atorvastatin can inhibit the inflammatory reaction initiated by toll like receptors 2 and 4, prevent monocyte aggregation, and effectively reduce the secretion of TNF-α, IL-1 and other inflammatory factors by macrophages.^17^–^19^ Khurana *et al*.^20^ also pointed out that atorvastatin can regulate the expression of microRNAs and peroxisome proliferator activated receptors to reduce the degree of inflammatory reaction in vivo. The results of our study showed that the serum levels of TNF-α, IL-1 and IL-6 in the combination group were lower than those in the Irbesartan group after the treatment, which further confirmed that the combination of atorvastatin and irbesartan can effectively reduce the micro-inflammatory state and may, therefore, have a higher application value in early DN.

In addition, the present study showed that the renal fibrosis indicators were significantly lower in the combined group compared to Irbesartan group. Studies have reported that Atorvastatin can help to improve renal fibrosis and restores renal function,^21^,^22^ and we speculate that the lower levels of renal fibrosis indicators in the combined group was because of the effect of Atorvastatin.

### Limitations:

This is a single-center retrospective study with a certain selection bias in the sample size. Additionally, indicators on blood glucose and lipid levels such as 2-h plasma glucose, fasting plasma glucose, triglyceride, total cholesterol, and low-density lipoprotein were not compared after treatment between the groups. A prospective cohort study with larger sample sizes and longer follow up is required to verify the conclusions of our analysis.

## CONCLUSION

Compared with Irbesartan alone, a combined Atorvastatin-Irbesartan regimen is more effective in the treatment of early DN. It more efficiently reduces the micro-inflammatory state of the body, improving renal function and fibrosis. Combined treatment is not associated with the increased risk of adverse reactions.

### Authors’ contributions:

**XZ:** Conceived and designed the study.

**XZ** and **JF:** Collected the data and performed the analysis.

**XZ:** Was involved in the writing of the manuscript and is responsible for the integrity of the study.

All authors have read and approved the final manuscript.
